# Supplementary material for: From Genetic Engineering to Preclinical Safety: A Study on Recombinant Human Interferons
Source: Int J Mol Sci. 2025 Dec 12;26(24):11982. doi: 10.3390/ijms262411982 (PMC12732355; doi:10.3390/ijms262411982)
Supplement: Supplementary file 1 [file ijms-26-11982-s001.zip › ijms-3939589-supplementary.pdf]

Supplementary information

From Genetic Engineering to Preclinical Safety: A Study on Recombinant Human Interferons

Thelvia I. Ramos [1,*], Carlos A. Villacis-Aguirre [2], Emilio Lamazares [2], Viana Manrique-Suárez [2], Felipe Sandoval [2], Cristy Culqui [3], Sarah Martin-Solano [1], Rodrigo Mansilla [4,] Ignacio Cabezas [5], Oliberto Sánchez [4], Sergio Donoso-Erch [5], Natalie C. Parra [2], María A. Contreras [2] and Nelson Santiago-Vispo [6].

1 Grupo de Investigación en Sanidad Animal y Humana (GISAH), Departamento de Ciencias de la Vida y la Agricultura, Universidad de las Fuerzas Armadas ESPE, Sangolquí 171103, Ecuador; ssmartin@espe.edu.ec

2 Biotechnology and Biopharmaceutical Laboratory, Departamento de Fisiopatología, Facultad de Ciencias Biológicas, Universidad de Concepción, Víctor Lamas 1290, P.O. Box 160-C, Concepción 4030000, Chile; carlovillacis@udec.cl, elamazares@udec.cl, vmanrique@udec.cl, felisandoval@udec.cl, natparra@udec.cl; mcontrerasv@udec.cl

3 Carrera Ingeniería en Biotecnología, Departamento de Ciencias de la Vida y la Agricultura, Universidad de las Fuerzas Armadas—ESPE, Sangolquí 171103, Ecuador; cnculqui@espe.edu.ec

4 Laboratory of Recombinant Biopharmaceuticals, Departamento de Farmacología, Facultad de Ciencias Biológicas, Universidad de Concepción, Víctor Lamas 1290, P.O. Box 160-C, Concepción 4030000, Chile; romansilla@udec.cl, osanchez@udec.cl

5 Clinical Sciences Department, Faculty of Veterinary Sciences, Universidad de Concepción, Vicente Méndez 595, Chillán 3780000, Chile; oscabeza@udec.cl, sedonoso@udec.cl

6 Clinical Biotec. Madrid 28029. Spain; santiago@clinicalbiotec.com

* Correspondence: tiramos@espe.edu.ec

Stability under Accelerated Conditions of rhIFNα-2b and rhIFN-γ.

Table S1. Accelerated stability of rhIFNα-2b, titer values, specific activity, and concentration.

Recombinant protein	Log EC_{50}	rhIFNα- 2b titer (IU/mL)	rhIFNα-2b specific activity (IU/mg)
Standard rhIFNα-2b	3.515	1.2×10^4	
rhIFNα-2b at 4 °C	2.126	8.7×10^3	2.8×10^7
rhIFNα-2b at 16 °C	2.794	9.5×10^3	2.8×10^7
rhIFNα-2b at 25 °C	2.720	9.3×10^3	2.8×10^7
rhIFNα-2b at 30 °C	2.507	8.5×10^3	2.6×10^7
rhIFNα-2b at 37 °C	3.213	1×10^4	3.3×10^7

The results demonstrate the antiviral activity of the protein obtained, evaluated at different temperatures under accelerated conditions.

Table S2. Accelerated stability of rhIFN-γ, titer values, specific activity, and concentration.

Recombinant protein	Log EC_{50}	rhIFN-γ titer (IU/mL)	rhIFN-γ specific activity (IU/mg)
Standard rhIFN-γ	2.202	1.1×10^7	6.64×10^{10}
rhIFN-γ at 4 °C	3.394	1.6×10^7	2.2×10^{11}
rhIFN-γ at 16 °C	2.295	1.1×10^7	1.5×10^{11}
rhIFN-γ at 25 °C	2.009	1.0×10^7	1.3×10^{11}
rhIFN-γ at 30 °C	2.578	1.2×10^7	1.7×10^{11}
rhIFN-γ at 37 °C	1.344	6×10^6	8.9×10^{10}

The results demonstrate the antiviral activity of the protein obtained, evaluated at different temperatures under accelerated conditions.

Subchronic Toxicity Assays with rhIFNα-2b in Rats.

Table S3. Subchronic toxicity. Blood chemistry of animals after 28 days of treatment.

Dose (IU/kg)	Hemoglobin	Hematocrit	GOT	GPT	Proteins	Urea	Cholesterol
0	16 ± 1.9	**59 ± 2.6**	**78.8 ± 5.2**	8.0 ± 1.043	**139.91 ± 3.77**	12.66 ± 2.60	2.26 ± 0.147
1 x 10^5	16.3 ± 0.6	**51 ± 5.6**	**50.4 ± 4.4**	5.46 ± 1.33	**96.41 ± 8.64**	14,68 ± 1.19	1.89 ± 0.188
1 x 10^6	16.1 ± 0.56	**57 ± 2.8**	**59.1 ± 5,35**	4.5 ± 0,38	**96.8 ± 7.87**	14.80 ± 0.75	2.49 ± 0.072
3 x 10^6	18 ± 0.2	**57 ±**	**55.1 ± 4.32**	6.0 ± 1.46	**113 ± 9.16**	20.58 ± 1.80	2.44 ± 0.15

Treatment groups were compared using a Student's t-test and Mann Whitney test. The statistical significance was set at α = 0.05.

Table S4. Subchronic toxicity. Blood chemistry of animals 28 days post-treatment.

Dose (IU/kg)	Hemoglobin	Hematocrit	GOT	GPT	Proteins	Urea	Cholesterol
0	17.0 ± 0.6	**43.0 ± 1.2**	**19.0 ± 4.7**	4.9 ± 0.97	**179.6 ± 8.13**	15.0 ± 2.16	2.08 ± 0.258
1 x 10^5	16.5 ± 0.6	**51.1 ± 1.7**	**19 ± 0.2**	7.8 ± 0.43	**136.8 ± 8.27**	5.93 ± 0.97	1.15± 0.043
1 x 10^6	16.1 ± 1.76	**47.0 ± 0.7**	**19.0 ± 3.7**	6.7 ±1.67	**139.0 ± 7.2**	10.04 ± 0.23	1.18 ± 0.97
3 x 10^6	18.0 ± 0.5	**44.0 ± 1**	**19.0 ± 6.3**	6.0 ± 1.15	**155.6 ± 11.34**	11.41 ± 2.41	2.11 ± 0.145

Treatment groups were compared using a Student's t-test and Mann Whitney test. The statistical significance was set at α = 0.05.

Pyrogen study.

Table S5. Pyrogen Study. Temperature changes.

Weight (g) / Treatment		Group I	Group II	Group III	Group IV	Group V	ANOVA F (p)
**Total**		**2**	**2**	**2**	**2**	**2**	
Start / Mean ± SD		37.0 ± 0.5	38.2 ± 0.1	37.5 ± 0.7	38.1 ± 0.1	38.6 ± 0.8	**2.495 (0.172)**
End / Mean ± SD		37.5 ± 0.4	37.0 ± 0.9	37.8 ± 0.7	37.9 ± 0.6	**36.8 ± 0.7***	**0.88 (0.536)**
**p (Student's t)**	Start vs End	0.07	0.28	Indefinite t No variability	0.61	**0.035***	

The table represents the mean ± SD. The treatment groups were compared through a one-way ANOVA and a Student's t-test for dependent samples. The statistical significance was set at α = 0.05.

Table S6. Pyrogen study. Temperature fluctuations in the animals in the study.

Animal	Group	Start	End	Fluctuations
C	1	36.7	37.2	0.50
F	1	37.4	37.8	0.40
A	2	38.3	37.7	-0.60
E	2	38.1	36.4	-1.70
H	3	37.0	37.3	0.30
I	3	38.0	38.3	0.30
G	4	38.2	38.3	0.10
J	4	38.0	37.4	-0.60
B	5	39.2	37.3	-1.90
D	5	38.0	36.3	-1.70

Safety Study of the Active Ingredients of rhIFNα-2b-rhIFN-γ in Sheep.

Table S7. Study in a Higher Organism (Sheep). Weight Behavior.

**Weight (g) / Treatment**		**Group I**	**Group II**	**Group III**	**Group IV**	**ANOVA F (p)**
**Total**		**4**	**4**	**4**	**4**	
Start / Mean ± SD		44.1 ± 6.3	53.0 ± 3.6	49.3 ± 9.2	48.0 ± 5.5	1.278 (0.396)
End / Mean ± SD		45.6 ± 5.9	54.7 ± 2.8	51.4 ± 9.9	49.8 ± 5.1	1.83 (0.195)
**p (Student's t)**	Start vs End	**0.013**	**0.026**	**0.01**	**0.006**	

Data are expressed as mean ± SD. The treatment groups were compared using repeated measures ANOVA. Since there were more than two evaluations, the Bonferroni error correction was considered. Additionally, a dependent samples Student's t-test was performed. The set statistical significance was $\alpha = 0.05$.

Table S8. Study in a Higher Organism (Sheep). Average Temperature During the Study.

**Temperature (°C) / Treatment**		**Group I**	**Group II**	**Group III**	**Group IV**	**ANOVA F (p)**
**Total**		**4**	**4**	**4**	**4**	
Week 1 / Mean ± SD		39.25 ± 0.17	39.40 ± 0.42	39.05±0.78	39.30 ± 0.18	0.525 (0.673)
Week 2 / Mean ± SD		39.10 ± 0.22	39.18 ± 0.32	39.08 ± 0.22	39.30 ± 0.18	0.705 (0.567)
Week 3 / Mean ± SD		39.00 ± 0.22	38.93 ± 0.28	38.98 ± 0.29	38.53 ± 0.92	0.760 (0.538)
Week 4 / Mean ± SD		39.23 ± 0.15	39.30 ± 0.18	38.98 ± 0.30	39.18 ± 0.10	2.005 (0.167)
**p (Student's t)**	Week 1 vs Week 4	0.638	0.495	0.848	0.141	

Treatment groups were compared using repeated measures ANOVA. When considering more than two evaluation times, the Bonferroni error correction was considered. For the pair-wise correlation in each treatment group, the dependent samples Student's t-test was performed. The set statistical significance was $\alpha = 0.05$.

Summary

Table S9. Comparative summary of the main functional and safety characteristics of rhIFNα-2b and rhIFN-γ.

**Dimension evaluated**	**rhIFNα-2b**	**rhIFN-γ**
**Specific activity**	High antiviral activity measured in a CPE assay (HEp-2 / Mengo).	Lower comparative antiviral activity in amplitude, but stable.
**Antiproliferative effect (HeLa)**	Significant reduction in cell viability in a dose-dependent curve.	Significant reduction in cell viability; comparable effect in the high range.
**Immunomodulatory effects**	This MHC-II activity is not described for rhIFNα-2b	Clearly and dose-dependently induces HLA-DR expression, consistent with immune activation.
**Accelerated stability (4–37 °C)**	Specific activity maintained without significant loss.	Specific activity maintained without significant loss.
**Endotoxins (LAL)**	Levels below the established regulatory limit.	Levels below the established regulatory limit.
**Acute toxicity (rodents)**	No mortality at doses 100-300 times higher than the estimated therapeutic dose.	No mortality at equivalent doses; no severe behavioral signs.
**Cardiorespiratory toxicity (rats)**	No mortality at doses 180 times higher than the estimated therapeutic dose.	No mortality at the evaluated doses; no severe behavioral signs of toxicity were recorded.
**Subchronic toxicity (28 days, rats)**	Transient changes (weight / biochemistry), with complete recovery after a drug-free period.	No relevant alterations observed in clinical or histopathological parameters.
**Pyrogen test (rabbits)**	Negative (no elevation ≥ 0.6°C).	Negative (no elevation ≥ 0.6°C).
**Mucosal safety (intranasal route, sheep)**	No significant histological lesions or inflammation.	No lesions; comparable tolerance.
